# 肺癌患者阿片类药物应用现状与合理性分析：单中心回顾性研究

**DOI:** 10.3779/j.issn.1009-3419.2020.102.12

**Published:** 2020-04-20

**Authors:** 雯娟 孙, 扬 胡, 玮 左, 波 张, 燕 徐

**Affiliations:** 1 100730 北京，北京协和医院药剂科 Department of Pharmacy, Peking Union Medical College Hospital, Beijing 100730, China; 2 100730 北京，呼吸与危重症医学科 Department of Respiratory and Critical Care Medicine, Peking Union Medical College Hospital, Beijing 100730, China

**Keywords:** 肺肿瘤, 癌痛, 阿片药物, 限定日费用, 合理性, Lung neoplasms, Cancer pain, Opioids, Limited day fee, Reasonable

## Abstract

**背景与目的:**

癌痛严重影响患者体力活动、心理状态以及生活质量，甚至缩短患者生存时间，合理规范应用阿片类药物可有效控制癌痛。肺癌患者癌痛发病率高，需分析肺癌患者阿片类药物应用现状，以评估肺癌患者阿片类药物应用的合理性。

**方法:**

回顾性分析2018年6月-2019年6月北京协和医院呼吸与危重症医学科肺癌住院患者的临床资料，分析305例应用阿片类药物的肺癌癌痛患者的临床信息和用药信息。

**结果:**

应用阿片类药物的肺癌患者影响因素分析中，年龄与阿片药物应用种类为主要影响因素。男性比例高于女性，且男性患者应用阿片药物种类多、年龄多集中在60岁-69岁之间。我院肺癌患者应用阿片类药物品种和结构符合要求，用药频率最高的为羟考酮缓释片，限定日费用（defined daily cost, DDC）排名第一位同时序号比为1，同步性好。吗啡片和吗啡注射液用量低，肺癌患者整体爆发痛（breakthrough cancer pain, BTcP）控制良好。阿片类药物整体应用合理（93.4%），不合理项目主要为爆发痛处置和用药频率不合理以及超剂量问题。

**结论:**

应用阿片类药物的肺癌癌痛患者多为男性，老年男性癌痛更难于控制，需要加强关注。我院肺癌患者阿片类药物整体应用合理，但仍需关注BTcP处置、阿片类药物用药剂量和特殊人群用药等问题。

## Abstract (en)

**Background and objective:**

Cancer pain affect the physical activity, psychological state and quality of life seriously, and even shortened the survival time of patients. Rational and standard application of opioids could control cancer pain effectively. Since the high incidence of cancer pain in lung cancer patients, the status of opioids application should be analyzed to evaluate the rationality of analgesic application in lung cancer patients.

**Methods:**

The clinical data of 305 patients with lung cancer who were hospitalized in Department of Respiratory and Critical Care Medicine of Peking Union Medical College Hospital from June 2018 to June 2019 were analyzed retrospectively.

**Results:**

In the analysis of factors affecting the use of opioids in lung cancer patients, age and drug kinds were the main influencing factors. The proportion of male patients was higher than female. Moreover, male patients used more kinds of opioids and were mostly between 60 and 69 years old. The variety and structure of opioids used in lung cancer patients in our hospital met the requirements. Oxycodone hydrochloride prolonged-release tablet has the highest frequency of application, and defined daily cost (DDC) ranked the first with a serial number ratio of 1, which showed good synchronization. The quantity of morphine tablet and morphine injection were low, and the overall breakthrough cancer pain (BTcP) in lung cancer patients was well controlled. The overall application of opioids were reasonable (93.4%), and the unreasonable items mainly included the disposal of BTcP and the unreasonable frequency of opioids, as well as overdose.

**Conclusion:**

The majority of patients with lung cancer who take opioids are male, and the cancer pain in elderly men are more difficult to control, which needs to pay more attention. The overall application of opioids in the patients with lung cancer pain in our hospital are reasonable, but there are still some problems to focus, such as the disposal of BTcP, the dosage of opioids and the medication of special groups.

癌痛是肿瘤患者的常见症状，据世界卫生组织（World Health Organization, WHO）统计，全球每年新发恶性肿瘤约1, 000万，30%-50%伴有不同程度的癌痛^[1]^。初诊肿瘤患者癌痛发生率约25%，而晚期肿瘤患者的癌痛发生率为60%-80%，其中1/3为重度癌痛^[2, 3]^。肺癌是我国发病率和死亡率最高的恶性肿瘤^[4]^，多数肺癌患者在确诊时已处于晚期，合并骨转移、脑转移等，癌痛发生显著。加拿大一项17, 202例回顾性队列研究^[5]^提示，肺癌、妇科肿瘤、胃肠道肿瘤、泌尿系统肿瘤为应用阿片类药物排名前4位的瘤种；美国另一项826, 022例癌痛患者的大型队列研究^[6]^提示，肺癌不仅是应用阿片类药物最多的瘤种且癌痛发病率最高（19.4%）。本研究主要收集我院应用阿片类药物肺癌患者基本信息和用药信息，评估阿片类药物应用现状，分析用药特点及合理性。

## 1 资料与方法

### 1.1 临床资料收集

回顾性分析2018年6月-2019年6月我院呼吸与危重症医学科应用阿片类药物肺癌癌痛患者的基本信息和用药信息。数据来源于电子信息系统，包括患者病案号、性别、年龄，药品商品名、通用名、规格、给药途径、用法用量、用药天数、用药金额等。本研究为回顾性病例研究，不涉及伦理和患者知情同意。

### 1.2 纳入标准

（1）诊断肺癌合并疼痛患者；（2）应用阿片类药物的患者。排除标准：（1）不含癌痛诊断的肺癌患者；（2）非肺癌诊断应用阿片类药物的患者。本研究共纳入符合标准的肺癌癌痛患者305例。

### 1.3

限定日剂量（defined daily dose, DDD）、用药频度（defined daily doses, DDDs）、限定日费用（defined daily cost, DDC）、序号比计算按药品名称统计阿片类药物用量、DDDs、DDC和序号比。具体分析方法包括：DDD：采用2019年WHO推荐的限定日剂量统计值；DDDs=某药总消耗量/该药DDD值。DDDs主要衡量药物使用频率，值越大表明该药使用频率越高，反之则越低；DDC=某药总用药金额/该药DDDs，DDC可作为药品经济费用方面的指标，反映使用该药的平均日费用；序号比=某药用药金额排序序号/DDDs排序序号，序号比反映用药金额与DDDs的同步性。

### 1.4 评价电子处方合理性

主要评价内容为用药频率、用药剂量、爆发痛处置。癌性爆发痛（breakthrough cancer pain, BTcP）定义^[7]^：在背景痛控制相对稳定、镇痛药物充分应用的前提下，自发或在某些可预知或不可预知因素的诱发下突然出现的短暂疼痛加重。爆发痛处置剂量按每日背景阿片类镇痛剂量的10%-20%计算^[7]^。

### 1.5 统计学方法

应用SPSS 21.0统计软件进行数据处理，计量资料以均数±标准差（Mean±SD）表示或率（%）表示，组间比采用χ^2^检验，以*P* < 0.05为有统计学差异。

## 2 结果

### 2.1 患者年龄及性别分布

将患者按性别进行分组，分别对年龄、阿片药物用药种类、爆发痛处置情况进行统计计算，从而了解三种情况在男性或女性患者间是否存在统计学差异。分组情况及统计结果见表 1。

**1:** 应用阿片药物肺癌患者的影响因素分析 Analysis of influencing factors of opioids in lung cancer patients

Factor	Male	Female	*P*
Age (yr)			0.000, 16
< 40	5	4	
40-49	11	24	
50-59	46	29	
60-69	92	33	
70-79	26	34	
≥80	0	1	
Drug kind			0.02
1	142	96	
2	29	21	
3	7	6	
4	2	2	
BTcP			0.97
Yes	40	28	
No	140	97	
BTcP: breakthrough cancer pain.			

根据表 1，应用阿片类药物的305例患者中，男性占比59.0%，女性占比41.0%。统计结果显示，在男性组和女性组中，年龄和用药种类有统计学差异（*P* < 0.05），爆发痛处置无统计学差异（*P* > 0.05）。

### 2.2 阿片类药物用药品种

2018年6月-2019年6月我院肺癌癌痛患者应用阿片类药物共4种剂型，分别是缓释片剂、片剂、注射剂、贴剂。共10个品规，包括：羟考酮缓释片10 mg、羟考酮缓释片40 mg、芬太尼透皮贴剂4.2 mg、芬太尼透皮贴剂8.4 mg、可待因片30 mg、吗啡片5 mg、吗啡注射液10 mg、吗啡缓释片10 mg、吗啡缓释片40 mg、曲马多缓释片100 mg。其中，口服制剂7种，贴剂2种，注射剂1种。口服制剂的使用量（86.3%）高于贴剂（12.6%）和注射剂（1.2%）。各剂型中缓释片剂的使用量最高（84.7%），见表 2。

**2:** 不同剂型阿片类药物用量占比 Percentage of diferent opioids dosage form

Dosage form	*n*	Total quantity (mg)	%
Sustained release tablet	5	448, 280	84.7
Tablet	2	7, 701	1.5
Patch	2	66, 870	12.6
Injection	1	6, 411	1.2
Total	10	529, 262	100.0
Each drug was converted to the same dose intensity of morphine tablet according to the opioid dose-conversion coefficient table^[2]^, morphine (oral):tramadol (oral)=1:5; morphine (oral):oxycodone (oral)=(1.5-2.0):1; morphine (parenteral):morphine (oral)=1:3; morphine (oral):codeine (oral)=1:6.5; Fentanyl transdermal patch (*μ*g/h), *q72h*=1/2×oral morphine (mg), *qd*. Total dosage is the cumulative value of the converted dose intensity.			

### 2.3 阿片类药物的DDDs和DDC及排序

我院肺癌患者使用阿片类药物的DDDs、DDC和序号比见表 3。肺癌患者应用阿片类药物DDDs排名中，羟考酮缓释片10 mg、羟考酮缓释片40 mg和曲马多缓释片100 mg排名前3位；吗啡片5 mg、吗啡缓释片10 mg和吗啡缓释片30 mg排名后3位。羟考酮缓释片10 mg和40 mg的DDC排名前2位，且序号比均为1，同步性好。曲马多缓释片100 mg DDC排序第8位且序号比 > 1，经济性好。芬太尼透皮贴剂8.4 mg DDC排序第6位但且序号比 < 1，日均费用较高。吗啡片和吗啡注射液序号比为1，同步性较好。

**3:** 阿片类药物DDDs、DDC、序号比 DDDs, DDC, serial number of opiods

Drug name	Specification	DDD	DDDs	Rank	DDC	Rank	Serial number
Oxycodone hydrochloride prolonged-release tablet	10 mg	75 mg	1, 522.9	1	62.1	1	1.0
Oxycodone hydrochloride prolonged-release tablet	40 mg	75 mg	1, 068.3	2	59.59	2	1.0
Tramadol hydrochloride sustained-release tablet	100 mg	0.3 g	1, 073.7	3	12.39	8	1.3
Fentanyl transdermal patch	8.4 mg	1.2 mg	1, 022.0	4	17.84	6	0.8
Codeine phosphate tablet	30 mg	0.1 g	484.7	5	2.83	10	1.2
Fentanyl transdermal patch	4.2 mg	1.2 mg	278.3	6	21.44	5	0.8
Morphine hydrochloride injection	10 mg	30 mg	71.2	7	10.68	9	1.1
Morphine hydrochloride sustained-release tablet	30 mg	0.1 g	31.2	8	27.2	4	0.9
Morphine hydrochloride sustained-release tablet	10 mg	0.1 g	4.8	9	37.4	3	1.0
Morphine hydrochloride tablet	5 mg	0.1 g	2.5	10	15	7	1.0
DDDs: defined daily doses; DDC: defined daily cost; DDD: defined daily dose.							

### 2.4 阿片类药物应用合理性分析

305例肺癌患者中，阿片类药物应用超过2种的患者共67例，累积应用阿片类药物393例。分别从爆发痛处置、用药频率、用药剂量三个方面进行合理性分析，结果见表 4。阿片类药物合理用药占比为93.4%，芬太尼透皮贴剂、吗啡片和吗啡缓释片合理用药占比最高（100.0%），吗啡注射液合理性较低（79.4%）。68例患者出现爆发痛，爆发痛处置次数共129次。爆发痛处置不合理患者14例，累积23例次。阿片类药物不合理项目分析中，爆发痛处置不合理23例次，用药频率不合理共8例次，用药剂量不合理4例次；不合理用药分析中，爆发痛处置占比最高（65.7%），其次为用药频率（22.9%）。爆发痛处置中以低于最低处置量的项目最多，占比60.9%（14/23），具体见表 5。

**4:** 阿片类药物合理性分析 Rational analysis of opioids in lung cancer

Drug name	Specification	Reasonable number	Unreasonable number	Rational drug use (%)
Oxycodone hydrochloride prolonged-release tablet	10 mg	115	5	95.8
Oxycodone hydrochloride prolonged-release tablet	40 mg	26	2	92.9
Tramadol hydrochloride sustained-release tablet	100 mg	105	4	96.3
Fentanyl transdermal patch	8.4 mg	9	0	100.0
Codeine phosphate tablet	30 mg	46	1	97.9
Fentanyl transdermal patch	4.2 mg	9	0	100.0
Morphine hydrachloride injection	10 mg	54	14	79.4
Morphine hydrochloride sustained-release tablet	30 mg	1	0	100.0
Morphine hydrochloride sustained-release tablet	10 mg	1	0	100.0
Morphine hydrochloride tablet	5 mg	1	0	100.0
Total		367	26	93.4

**5:** 阿片类药物不合理用药类型分析 Unreasonable use analysis of opioids

Evaluation indicator	Unreasonable use of drug	*n*	%
BTcP	Above disposal* 1 mg-5 mg, 6 cases; Above disposal 5 mg-10 mg, 3 cases; Below disposal 1 mg-5 mg, 4 cases; Below disposal 5 mg-10 mg, 6 cases; Below disposal 10 mg-15 mg, 4 cases.	23	65.7
Frequency	Oxycodone hydrochloride prolonged-release tablet, 10 mg/120 mg *q8h*, 5 cases; Oxycodone hydrochloride prolonged-release tablet, 10 mg/20 mg *tid*, 2 cases; Tramadol hydrochloride sustained-release tablet, 50 mg *q6h*, 1 case.	8	22.9
Dose	Codeine phosphate tablet, 90 mg *tid*, 1 case; Tramadol hydrochloride sustained-release tablet, 400 mg *bid*, 1 case; Tramadol hydrochloride sustained release tablet, 200 mg *tid*, 2 cases.	4	11.4
BTcP disposal is calculated at 10 to 20 percent of daily dose of background opioids^[7]^, the disposal drug* is morphine injection.			

## 3 讨论

本研究305例应用阿片类药物的肺癌患者中，男性占比高于女性（59.0% *vs* 41.0%），与美国大型回顾性研究^[8]^中女性占比高于男性结果不同（55.7% *vs* 44.3%），但与中国肺癌男性患者发病率高于女性整体情况一致^[9]^。年龄 > 60岁的患者达61%，与美国大型回顾性研究^[6]^结果相同。对男女两组应用阿片药物肺癌患者的影响因素分析，结果显示男性患者年龄多集中在60岁-69岁之间且阿片类药物应用种类更多，研究提示老年男性癌痛更难于控制，需要加强关注。

我院肺癌患者应用阿片药物主要包括10个品种、4种剂型。口服制剂的使用频率显著高于其他剂型（86.3%），其中缓释片剂的使用率最高（84.7%），与WHO倡导的癌症患者止痛药物首选口服制剂的原则一致。癌痛治疗以口服制剂为主，缓控释制剂有助于维持稳定有效的血药浓度，是慢性癌痛患者的首选治疗方案。强阿片类药物临床应用最广，是癌痛治疗的主要手段。我院肺癌患者三阶梯药物用量整体高于二阶梯药物用量，且口服药物与外用药物的使用量始终高于注射剂，符合“首选无创途径给药”并弱化二阶梯药物的指导原则。

本研究305例患者中，肺癌癌痛患者中强阿片类药物使用频率最高，其中羟考酮缓释片的DDDs最高，吗啡片与吗啡缓释片最低。羟考酮缓释片是美国国家综合癌症网络（National Comprehensive Cancer Network, NCCN）临床实践指南推荐的中重度癌痛首选用药之一^[10]^。羟考酮缓释片属于强效合成镇痛药，速释与缓释部分（38%即释，62%缓释）结合，速释部分迅速止疼，缓释部分长效释放，同时其等效镇痛强度为吗啡的1.5倍-2.0倍，因此疼痛控制效果较好。羟考酮缓释片10 mg及羟考酮缓释片40 mg的DDC排名前2位，且序号比均为1，同步性好。曲马多缓释片为二阶梯止痛药物，DDDs排名第3位，用药频率较高且序号比 > 1，表明应用率高且价格较低可减轻患者负担。芬太尼透皮贴剂8.4 mg的DDDs位居第4位，DDC排名第6位但序号比 < 1，说明日均费用较高。芬太尼透皮贴剂特点为镇痛效果持久稳定且不良反应少而轻，尤其适用于口服阿片类药物胃肠道反应不耐受的癌痛患者，是癌痛患者重要的可选治疗途径之一。吗啡片和吗啡注射液主要用于疼痛滴定和爆发痛处置，我院肺癌患者吗啡片和吗啡注射液用量低，提示我院肺癌癌痛患者整体爆发痛发生率较低，整体控制良好。

本研究305例患者中，阿片类药物整体应用合理（93.4%），不合理项目主要为爆发痛处置不合理、用药频率不合理以及超剂量等。在癌痛患者中，BTcP的发生率可达33%-95%^[11]^。BTcP不仅严重影响患者的日常活动，导致生存质量、治疗依从性的下降，还会增加医疗资源的支出^[12]^，提示临床预后较差且容易发生阿片类药物抵抗^[3]^。BTcP为难治性癌痛，主要体现为疼痛多不可预测，病理机制复杂，解救药物均有滞后性^[7]^。本研究中，爆发痛处置不合理内容主要为处置剂量不足，其次为处置剂量超量。提示临床应加强剂量换算，关注BTcP处置剂量合理性。羟考酮缓释片不合理用药内容为给药频次增加，增加频率原因包括药物疼痛控制不足12 h和特殊情况用药。例如，1例67岁老年男性，左肺腺癌骨转移，2个周期化疗后，骨痛明显。羟考酮缓释片加量至100 mg *q12h*，夜间仍出现多次爆发痛，根据背景剂量和爆发痛处置量再次加量至羟考酮缓释片120 mg *q12h*后，患者出现谵妄等精神系统障碍。因非甾体抗炎药物会加重肝损及胃肠道反应，尝试80 mg *tid*给药。用药后患者疼痛可控，同时谵妄症状减轻。因此，对于特殊癌痛患者，如有特殊给药频率等临床需求，建议医生于处方上注明原因。曲马多缓释片不合理用药内容为超剂量给药和给药频率增加。肝肾功能正常患者，曲马多缓释片最大剂量不超过400 mg/d，老年患者不超过300 mg/d；肾功能异常患者，为降低癫痫发作的风险日剂量不超过200 mg^[10]^。曲马多是弱的μ受体激动剂，如按100 mg *q6h*给药，其镇痛效果仍低于吗啡等其他阿片类药物，还可能增加不良反应。因此建议用药频率间隔不少于8 h，如果疼痛无法控制建议换用强阿片类药物。可待因片不合理用药内容为超剂量给药。可待因是前体药物，经肝药酶CYP2D6代谢为吗啡，再经Ⅱ期代谢途径代谢为吗啡-6-葡萄糖醛酸内酯。CYP2D6在不同种族和个体之间存在多态性，低CYP2D6活性的个体服用可待因后镇痛效果降低或消失，高CYP2D6活性会快速产生大量代谢产物，引发吗啡中毒。可待因片极量为240 mg/d，因此必须监测可待因剂量安全限度。

我院肺癌患者阿片类药物品种、剂型符合临床需求，使用结构符合癌痛治疗原则。应用阿片类药物的肺癌患者多为男性，老年男性癌痛更难于控制，需要加强关注。我院肺癌癌痛患者阿片类药物整体应用合理，但仍需关注阿片类药物用药剂量、爆发痛处置和特殊人群用药等问题。随着我院“无痛病房”的发展，临床药师已逐渐开展医嘱审核、用药咨询、重点患者培训和监测等工作。患者教育内容包括医药护联合癌痛用药宣教、患者教育、癌痛药物应用注意事项培训等，目的在于推进阿片类药物的合理使用，服务临床。
